# Effects of Variable Ventilation on Gas Exchange in an Experimental Model of Capnoperitoneum: A Randomized Crossover Study

**DOI:** 10.1213/ANE.0000000000007418

**Published:** 2025-01-22

**Authors:** Álmos Schranc, Roberta Südy, John Daniels, Fabienne Fontao, Ferenc Peták, Walid Habre, Gergely Albu

**Affiliations:** From the 1 Unit for Anaesthesiological Investigations, Department of Anaesthesiology, Pharmacology, Intensive Care and Emergency Medicine, University of Geneva, Geneva, Switzerland; 2 Department of Medical Physics and Informatics, University of Szeged, Szeged, Hungary; 3 Pediatric Anesthesia Unit, Department of Anaesthesiology, Pharmacology, Intensive Care and Emergency Medicine, University Hospitals of Geneva, Geneva, Switzerland; 4 Division of Anaesthesiology, Department of Anaesthesiology, Pharmacology, Intensive Care and Emergency Medicine, University Hospitals of Geneva, Geneva, Switzerland.

## Abstract

**BACKGROUND:**

The rapid advancement of minimally invasive surgical techniques has made laparoscopy a preferred alternative because it reduces postoperative complications. However, inflating the peritoneum with CO_2_ causes a cranial shift of the diaphragm decreasing lung volume and impairing gas exchange. Additionally, CO_2_ absorption increases blood CO_2_ levels, further complicating mechanical ventilation when the lung function is already compromised. Standard interventions such as lung recruitment maneuvers or increasing positive end-expiratory pressures can counteract these effects but also increase lung parenchymal strain and intrathoracic pressure, negatively impacting cardiac output. The application of variability in tidal volume and respiratory rate during mechanical ventilation to mimic natural breathing has shown benefits in various respiratory conditions. Therefore, we aimed to evaluate the short-term benefits of variable ventilation (VV) on gas exchange, respiratory mechanics, and hemodynamics during and after capnoperitoneum, compared to conventional pressure-controlled ventilation (PCV).

**METHODS:**

Eleven anaesthetized rabbits were randomly assigned to PCV or VV. Oxygenation index (Pao_2_/FiO_2_), arterial partial pressure of carbon dioxide (Paco_2_), and respiratory mechanical parameters were assessed after a 15-minute-long ventilation period before, during, and after capnoperitoneum. According to a crossover design, after measurements at the 3 different stages, the ventilation mode was changed, and the entire sequence was repeated.

**RESULTS:**

Capnoperitoneum compromised respiratory mechanics, decreased oxygenation, and caused CO_2_-retention compared to baseline measurements under both ventilation modalities (*P* < .05, for all). Application of VV resulted in lower Pao_2_/FiO_2_ (405. 5 ± 34.1 (mean ± standard deviation [SD]) vs 370. 5 ± 44.9, *P* < .001) and higher Paco_2_ (48. 4 ± 5.1 vs 52. 8 ± 6.0 mm Hg, *P* = .009) values during capnoperitoneum compared to PCV. After abdominal deflation and a lung recruitment maneuver, VV proved more beneficial for CO_2_ removal than PCV (41. 0 ± 2.3 vs 44. 6 ± 4.3mmHg, *P* = .027). No significant difference was observed in the respiratory mechanical or hemodynamic parameters between the ventilation modalities under the same conditions.

**CONCLUSIONS:**

The detrimental effects of capnoperitoneum on gas exchange were more pronounced with VV. However, after the release of capnoperitoneum, VV significantly improved CO_2_ clearance. Therefore, VV could possibly be considered as an alternative ventilation modality to restore physiological gas exchange after, but not during, capnoperitoneum.

**KEY POINTS**

**Question:** Does variable ventilation prevent the adverse effects of capnoperitoneum on gas exchange and respiratory mechanics?

**Finding:** Variable ventilation showed inferior gas exchange during capnoperitoneum than conventional pressure-controlled ventilation. However, it increased CO_2_ clearance after abdominal deflation.

**Meaning:** Variable ventilation may be an alternative ventilation modality after abdominal deflation after capnoperitonium, to restore Paco_2_ to physiological levels. This modality may be of interest for patients undergoing long laparoscopic interventions especially with some level of respiratory disease, causing CO_2_ retention.

Due to the rapid advancement in minimally invasive techniques, laparoscopy has gained broad acceptance across diverse surgical domains.^1,2^ This approach reduces postoperative complications and enhances patient outcomes.^2–4^ However, laparoscopy necessitates the inflation of the peritoneum with CO_2_ to facilitate precise surgical manipulations.^5^ While capnoperitoneum enables such maneuvers, cranial shifting of the diaphragm decreases lung volume and subsequently deteriorates lung tissue mechanics and gas exchange.^6–8^ Moreover, peritoneal absorption contributes to an increase in the CO_2_ content of blood. This excess CO_2_ must be eliminated by the lungs, whose function may already be compromised.^2^ In such circumstances, intraoperative recruitment maneuvers^9^ or elevating positive end-expiratory pressure (PEEP) can be used to counterbalance the volume loss and the gas exchange impairment. Nevertheless, these interventions increase lung parenchymal strain, and the resultant elevated intrathoracic pressure jeopardizes venous return, subsequently worsening cardiac output (CO).^10^ Consequently, developing alternative ventilation modalities during laparoscopic surgery that minimizes stress and strain on the lung tissue is of paramount importance.

Variable ventilation (VV) as an alternative modality introduces variability into an otherwise monotonous breathing pattern by altering tidal volume (VT) and respiratory rate (RR) breath to breath mimicking physiological breathing.^11^ The introduction of variability has been proved to efficiently increase lung volume by reopening atelectatic areas and reducing further atelectasis development by a phenomenon called stochastic resonance.^12^ Reopening closed alveoli not only improves gas exchange by reducing shunt fraction but also stabilizes the parenchymal structure leading to better lung compliance allowing lower ventilation pressures causing less parenchymal injury. This has been proved to be beneficial during prolonged application of VV compared to conventional ventilation modalities in experimental models of chronic obstructive pulmonary disease,^13^ in lung fibrosis^14^ and in acute respiratory distress syndrome.^11,15–19^ In these respiratory pathologies where the ventilation scenario is challenged by atelectasis, lung inhomogeneity, airflow limitation and inflammation leading to impaired gas exchange.

The functional and structural impairments of the respiratory system during capnoperitoneum are the iatrogenic consequence of the intraabdominal CO_2_ inflation,^6–8^ resulting in CO_2_ retention due to atelectasis development and increased shunt fraction in the presence of an additional CO_2_ load. In this scenario, the sudden and dynamic decrease in respiratory compliance is only temporary and is limited to the duration of the capnoperitoneum and the immediate postoperative period. However, the potential short-term benefits of VV in this dynamically changing scenario have not yet been evaluated.

Thus, we aimed at characterizing the effects of VV on gas exchange, respiratory mechanics, and hemodynamics during and after capnoperitoneum. We also aimed at comparing these results to those measured using conventional pressure-controlled ventilation (PCV) in a prospective, randomized crossover experimental design. We hypothesized that the deterioration of gas exchange and lung mechanics during capnoperitoneum may be prevented with the application of breath-by-breath variation in VT and RR.

## METHODS

### Study Design

The experimental protocol was approved by the Animal Welfare Committee of the Canton of Geneva and the Experimental Committee of the University of Geneva, Switzerland (no. 34560/GE183A, dated February 16, 2021). All procedures were performed in accordance with current Swiss animal protection laws (LPA, RS455). The current report follows the Animal Research: Reporting of In Vivo Experiments (ARRIVE) guidelines.^20^ Eleven New Zealand White, male rabbits (3.9 ± 0.2 kg; mean ± standard deviation [SD]) were purchased (Charles River Laboratories) and delivered at least 7 days before the experiments to allow acclimatization. The rabbits had access to food and water ad libitum before the experiments.

### Anesthesia and Surgical Preparation

The animals were premedicated with intramuscular ketamine (25 mg/kg; Labatec-Pharma SA, Product #DIS12086) and xylazine (3 mg/kg; Elanco, Product #VETO109AA309U). Fifteen minutes later, an ear vein was cannulated (22-G Abbocath-T, Abbott-Hospira, Product #G719-A01). A surgical tracheostomy using a 3.5-mm uncuffed tube (Covidien, Product #9335E) was performed after infiltration of the anterior cervical region with lidocaine 1% (Sintetica, Product #104000030). Anesthesia was maintained with continuous intravenous propofol 2% (10 mg·kg^−^^1^·h^−^^1^; Fresenius Kabi, Product #4282051), fentanyl (5 µg·kg^−^^1^·h^−^^1^; Mepha Pharma, Product #320897), and midazolam (0.2 mg·kg^−^^1^·h^−^1; Sintetica, Product #101000113). After ensuring adequate levels of anesthesia and analgesia, neuromuscular blockade was performed using continuous intravenous atracurium (0.6 mg·kg^−^^1^·h^−^^1^; Labatec-Pharma SA, Product # DIS12011). The right jugular vein and the left femoral artery were cannulated with 22-G catheters (Abbocath-T, Abbott-Hospira, Product #G719-A01). Body temperature was measured through a rectal thermometer and maintained at 38°C–39°C using a thermostatic heating pad (Harvard Apparatus). To mimic the clinical scenario of a laparoscopic surgery, an intraabdominal trocar (Covidien Versaport Bladeless, Medtronic Schweiz AG) was surgically introduced and fixed with a running suture to ensure a complete seal. The ports of the trocar were closed until the abdomen was insufflated with CO_2_ during the appropriate protocol stages, which are detailed below.

### Mechanical Ventilation

A modified version of the previously described custom-made blower-driven ventilator^21^ with a closed breathing circuit was used to deliver conventional PCV. A custom-made software was used to control the rotation speed of the blower and generate the required changes in the airway pressure pattern for the different mechanical ventilation modalities. During PCV, the constant rotation speed of the blower was altered periodically to provide the required respiratory rate and VT. VV was performed using breath-by-breath variation in RR and in peak inspiratory pressures (PIP), consequently in VT based on a previously established pattern signal.^13^ During VV, PIP and RR variability was set to target the same VT and RR values averaged over 30 breath cycles as set with PCV.

### Assessment of Gas Exchange

Arterial and central venous blood samples (0.15 mL) were collected and analyzed simultaneously (VetScan i-STAT1, Abaxis) to determine the arterial Po_2_ (Pao_2_), carbon dioxide (Paco_2_) and the intrapulmonary shunt fraction (Qs/Qt). The capillary (CcO_2_), arterial (Cao_2_), and venous (CvO_2_) oxygen contents were calculated as follows:

Cao_2_ = 1.34·Hb_art._·Sao_2_+Pao_2_·0.0031

CvO_2_ = 1.34·Hb_ven._·SvO_2_+PvO_2_·0.0031

CcO_2_ = 1.34·Hb_art._+PAO_2_·0.0031, where PAO_2_ = (FiO_2_(P_atmos_–P_H2O_))–(Paco_2_·0.8^–1^).

Then the modified Berggren equation was used to assess Qs/Qt^22^:


$$\begin{array}{l} \frac{Q_{s}}{Q_{t}}=\frac{CcO_{2}-CaO_{2}}{CcO_{2}-CvO_{2}}. \end{array}$$

### Assessment of Respiratory Mechanics

Respiratory oscillometry was used to measure the airway and respiratory tissue mechanical parameters as detailed previously.^21^ Briefly, the custom-made blower-driven ventilator generated a small amplitude pseudorandom forcing signal, which had 23 noninteger multiple frequency components between 0.5 Hz and 20.75 Hz during short apneic periods (8 sec) and was interposed into the mechanical ventilation. Oscillatory airflow (V′) was measured with a screen pneumotachograph (11-mm ID, PNT3500; Hans Rudolph, Inc) coupled with a differential pressure sensor (HCLA02X5B; First Sensor). Airway opening pressure was detected with a differential pressure transducer (HCLA0075B; First Sensor). The input impedance spectra of the respiratory system (Z_rs_) were calculated as Z_rs_ = P_ao_/V′. The input impedance of the tracheal tube and the connecting tubing was measured after the experiments and this was subtracted from the Z_rs_ spectra before the analyses.

**Figure 1.**
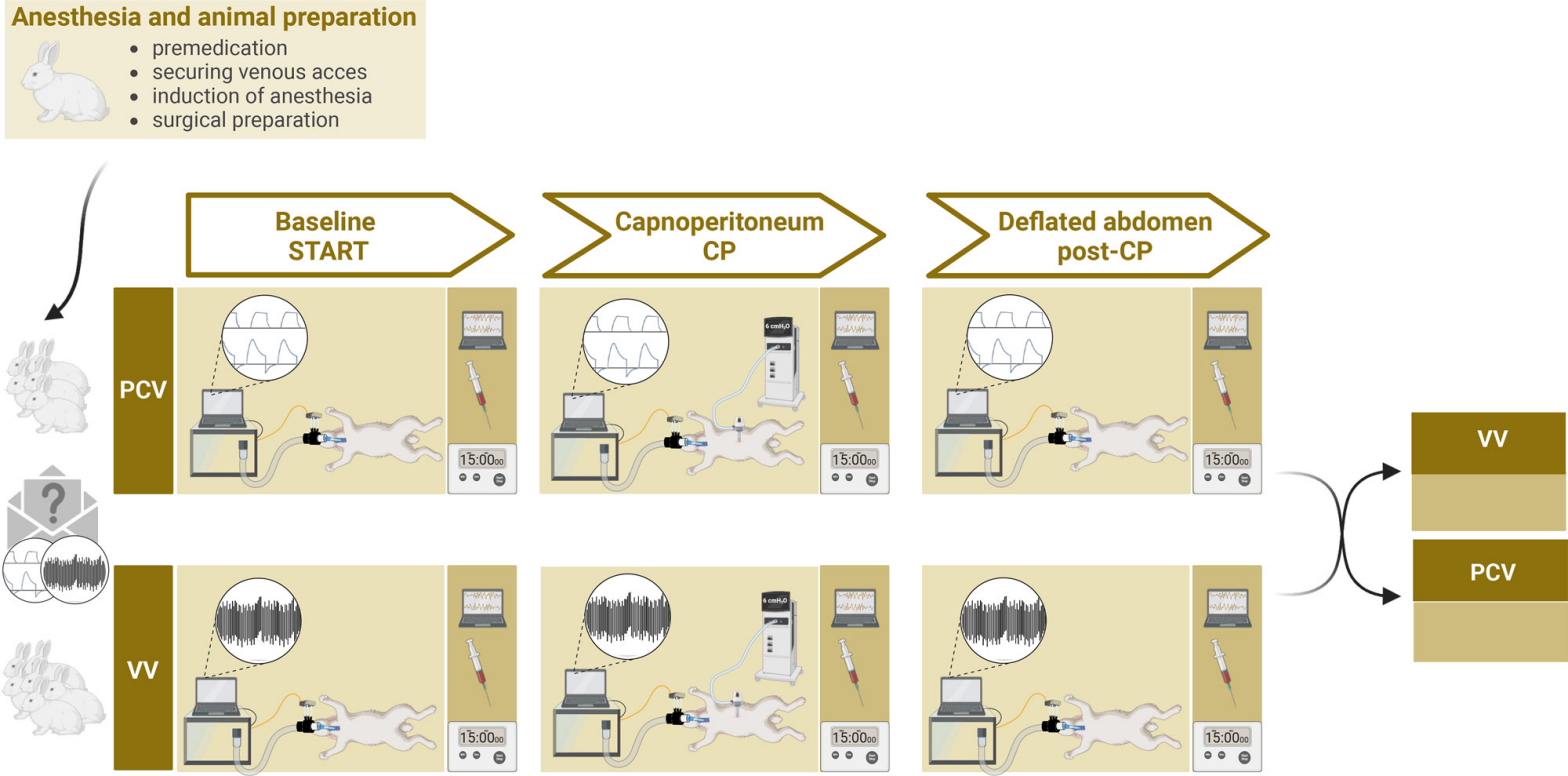
Schematic representation of the experimental protocol. CP, 15 min of ventilation in the presence of capnoperitoneum; PCV, pressure-controlled ventilation; post-CP, 15 min of ventilation after the deflation of the abdomen and a recruitment maneuver; START, 15 min of baseline ventilation; VV, variable ventilation.

The mechanical properties of the total respiratory system were characterized by fitting a well-validated constant phase model^23^ to the ensemble-averaged Zrs spectra under each experimental condition. The model comprised the frequency-independent airway resistance (R_aw_) and airway inertance in series and had a viscoelastic constant phase tissue component that incorporated tissue damping (G) and elastance (H).^24^ The tissue hysteresivity (η), which characterized the coupling between the dissipative and elastic forces within the respiratory tissues, was calculated as η = G/H.^25^

### Assessment of Hemodynamic and Ventilation Parameters

Mean arterial pressure (MAP), heart rate, and CO were continuously registered by PiCCO (PiCCO Plus, Pulsion Medical Systems). Ventilation parameters such as PIP, VT, and RR were determined by the ventilator. Driving pressure (P_driving_) was calculated as the difference between PIP and PEEP.

### Study Protocol

A schematic representation of the study protocol is presented in Figure 1. After the surgical preparation and a hyperinflation maneuver (using a sustained PIP of 25 cmH_2_O for 10 seconds) to standardize volume history, the animals were randomly assigned to 15 minutes of PCV (PIP set to target a VT of 7 mL/kg; RR: 20–25/min; PEEP: 5 cmH_2_O; fraction of inspired oxygen [FiO_2_] of 0.4; inspiratory to expiratory time ratio [I:E]: 1:2) or VV (PIP set to target an averaged VT of 7 mL/kg; an averaged RR: 20–25/min; PEEP: 5 cmH_2_O; FiO_2_: of 0.4; I:E ratio: 1:2). A set of initial data (stage START) was then collected. Subsequently, capnoperitoneum was initiated through an intraabdominal trocar using a laparoscopic insufflator to reach an intraabdominal pressure of 6 cmH_2_O. After 15 minutes, another set of data was collected (stage CP). The abdomen was then deflated, followed by a lung recruitment maneuver. Finally, the animals were ventilated for 15 minutes, and a last set of data was collected (stage post-CP). According to the crossover study design, the ventilation modality was then changed, and after a recruitment maneuver the entire data collection sequence was repeated. After completing the experiments, the animals were euthanized by an overdose of sodium pentobarbital (120 mg/kg).

### Primary Outcome Parameters

The primary outcome of the present study was the Pao_2_/FiO_2_ ratio.

### Secondary Outcomes

The following secondary outcomes were assessed:

- Gas exchange parameters: Paco_2_, and Qs/Qt.
- Respiratory mechanical parameters: Raw, G, H, and η.
- Hemodynamic parameters: MAP, CO, and HR.
- Ventilation parameters: P_driving_, RR, and VT.

### Exclusion Criteria

All the experimental animals were included in the final data analysis.

### Sample Size Estimation

The main question of the present study was to compare the efficiency of VV and PCV during capnoperitoneum. Therefore, answering our research question, the sample size calculation was based on the differences of primary outcome in terms of ventilation modalities during capnoperitoneum. Based on the results of our previous study in a similar model,^26^ an improvement of 25% in the primary outcome (Pao_2_/FiO_2_) with a 10% coefficient of variation can be anticipated when a VV modality is applied. We estimated the sample size based on a 2-way repeated measures analysis of variance (ANOVA), which showed that at least 10 animals were required to detect statistically significant changes with a statistical power of 0.8 and a 2-sided alpha error of 0.05 (GPower3 software). Considering the potential drop-out rate of approximately 10%, we included 11 animals.

### Statistical Analyses

Data are presented as mean ± SD. The relative changes regarding CP versus START and post-CP versus START are expressed as mean difference (∆_START_), and [95% confidence interval]. The Shapiro-Wilk test was used to test normality, and the equal variance was tested with the Brown-Forsythe test. Since the used statistical software assumes sphericity when ANOVA is performed, ensuring that no violation of sphericity was involved we applied Mauchly’s test, and for variables where sphericity was not met, we applied Huynh-Feldt and Greenhouse-Geisser corrections. Two-way repeated measures ANOVA with factors of ventilation modalities (PCV and VV) and the different stages (START, CP, and post-CP), along with their interactions, was used to test differences. Pairwise comparisons were performed by using Holm–Šidák post hoc analyses. To assess the correlation between capnoperitoneum-induced relative changes in respiratory tissue elastance and Pao_2_/FiO_2_, Pearson correlation analysis was performed. Statistical analyses were conducted with a significance level of *P* < .05, and all reported p values are 2-sided. The statistical tests were performed with SigmaPlot (version 15, Systat Software, Inc).

## RESULTS

**Figure 2.**
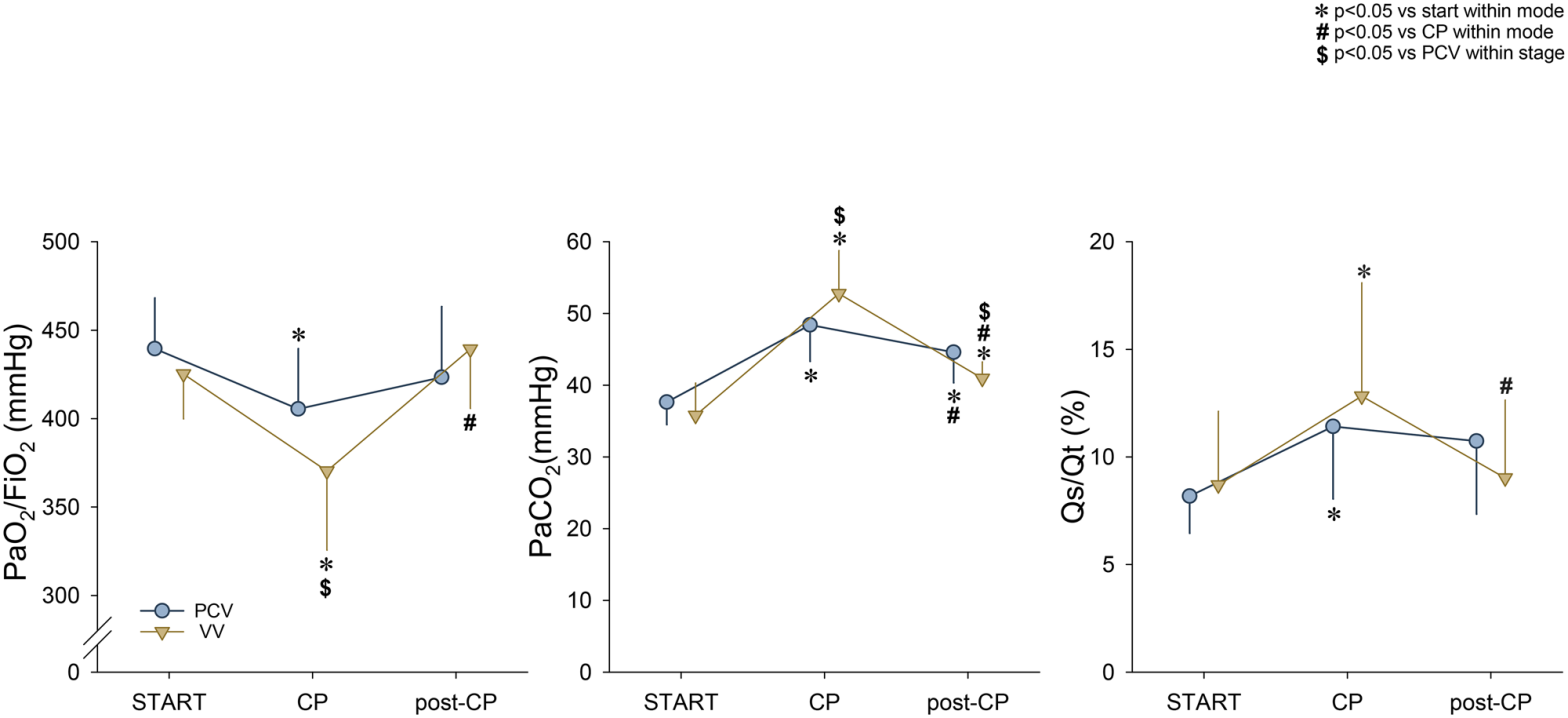
Gas exchange parameters during PCV and VV are expressed as mean ± SD. CP indicates 15 min of ventilation in the presence of capnoperitoneum; Pao_2_/FiO_2_, oxygenation index; Paco_2_, arterial partial pressure of carbon dioxide; PCV, pressure-controlled ventilation; post-CP, 15 min of ventilation after the deflation of the abdomen and a recruitment maneuver; Qs/Qt, intrapulmonary shunt fraction; SD, standard deviation; START, 15 min of baseline ventilation; VV, variable ventilation. *P < .05 vs START within a ventilation mode, #*P* < .05 vs CP within a ventilation mode; $*P* < .05 vs PCV within stage.

Gas exchange parameters are summarized in Figure 2 across different stages of the experiment. Capnoperitoneum significantly reduced Pao_2_/FiO_2_ (PCV – Δ_START_: −8% [−13% to 1%], *P* = .002; VV – Δ _START_: −13% [−29% to 3%], *P* < .001) and elevated Paco_2_ (PCV – Δ _START_: 28% [15%–51%], *P* < .001; VV – Δ _START_: 49% [21%–82%], *P* < .001) under both ventilation modes. Fifteen minutes after releasing the capnoperitoneum, Pao_2_/FiO_2_ (PCV – Δ_START_: −4% [−19% to 3%], *P* = .117; VV – Δ _START_: 3% [−6% to 9%], *P* = .137) levels returned to baseline while Paco_2_ remained elevated (PCV – Δ _START_: 19% [2%–50%], *P* = .015; VV – Δ _START_: 15% [3%–39%], *P* = .001), independent of the ventilation modality. During abdominal insufflation, Paco_2_ was higher (*P* = .009) and Pao_2_/FiO_2_ was lower (*P* = .001) under VV compared to PCV. However, postcapnoperitoneum, significant improvements in lung oxygenation and CO_2_ elimination were evidenced when applying VV (*P* < .001 for both). The intrapulmonary shunt fraction increased under both VV (Δ_START_: 61% [−35% to 336%], *P* < .001) and PCV (Δ_START_: 32% [−35% to 70%], *P* = .002) during capnoperitoneum, with significant decrease from these elevated levels only under VV after capnoperitoneum was released (*P* < .001). During post-CP, intrapulmonary shunt did not show a significant difference compared to START (PCV – Δ _START_: 26% [−20% to 97%], *P* = .165; VV – Δ _START_: 5% [−30% to 75%], *P* = .081). No significant difference was observed between the ventilation modalities at any stage (*P* > .05).

**Figure 3.**
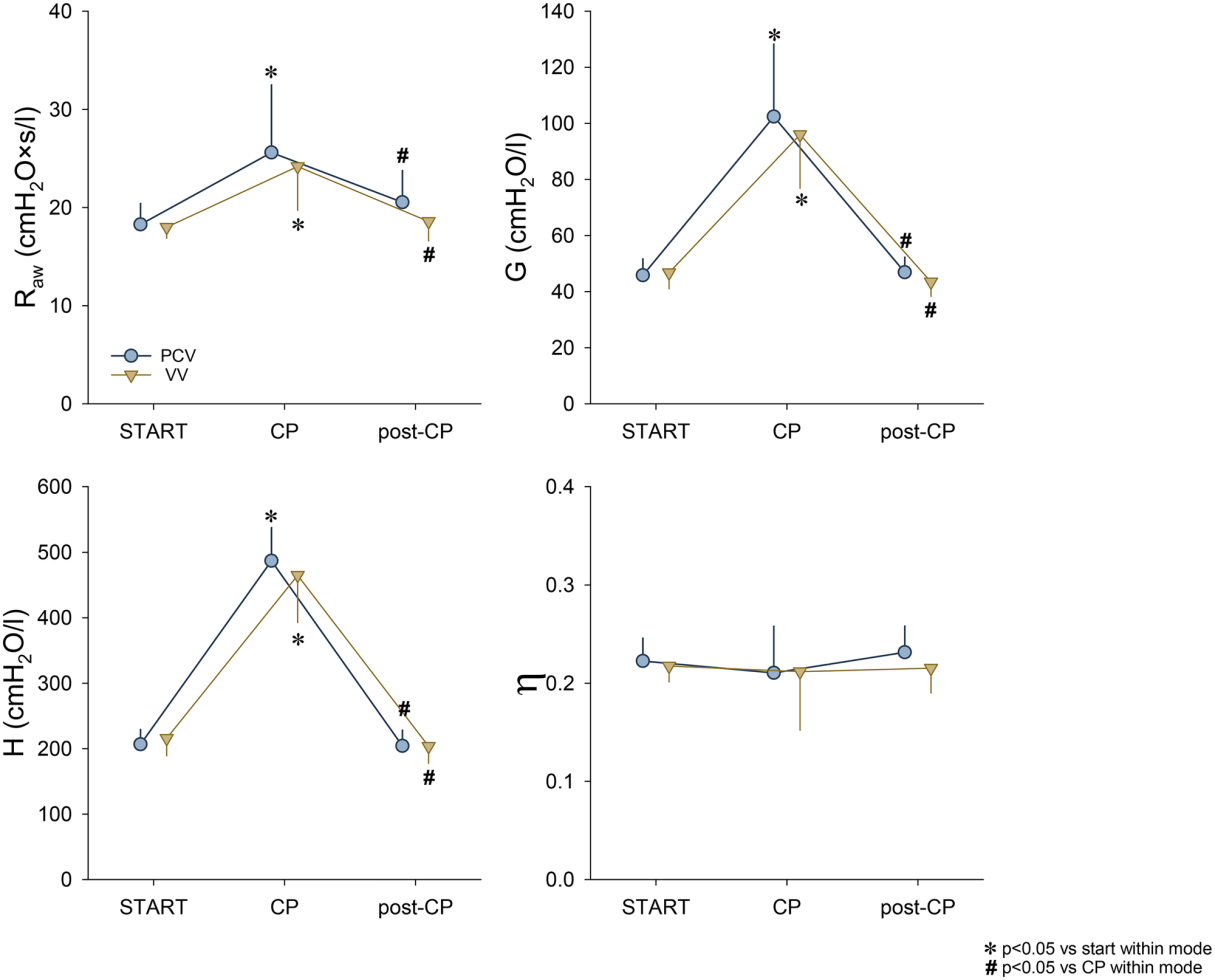
Respiratory mechanical parameters during PCV and VV are expressed as mean ± SD. CP indicates 15 min of ventilation in the presence of capnoperitoneum; G, respiratory tissue damping; H, respiratory tissue elastance; η, hysteresivity; PCV, pressure-controlled ventilation; post-CP, 15 min of ventilation after the deflation of the abdomen and a recruitment maneuver; R_aw_, airway resistance; START, 15 min of baseline ventilation; VV, variable ventilation. *P < .05 vs START within a ventilation mode, #*P* < .05 vs CP within a ventilation mode.

Figure 3 depicts the respiratory mechanical data derived from the oscillometric measurements at 3 different protocol stages. Capnoperitoneum significantly worsened every measured mechanical parameter (*P* < .001 for all). In the post-CP period, there was a complete recovery in all respiratory mechanical indices. The parameter η was unaffected by any interventions or ventilation modalities.

Hemodynamical and ventilation parameters are presented in the Table. Regarding systemic hemodynamics, capnoperitoneum elevated MAP (PCV – Δ _START_: 16% [−18% to 65%], *P* = .05; VV – Δ _START_: 18% [−22% to 50%], *P* = .016), while CO (PCV – Δ _START_: 16% [−17% to 42%], *P* = .06; VV – Δ _START_: 19% [−6% to 47%], *P* = .065) and HR (PCV – Δ _START_: −1.5% [−20% to 12%], *P* = .43; VV – Δ _START_: 3% [−12% to 6%], *P* = .67) remained unchanged. In the post-CP period, MAP decreased, and no significant difference was observed compared to baseline (PCV – Δ _START_: 15% [−15% to 67%], *P* = .05; VV – Δ _START_: 11% [−17% to 62%], *P* = .016). As for the ventilation parameters, while average VT and RR remained constant, P_driving_ increased during capnoperitoneum under both ventilation modes. In the post-CP phase P_driving_ returned to baseline.

**Table.** Hemodynamically and Ventilation Parameters Obtained During PCV and With VV, Shown As Mean ± SD

		MAP (mm Hg)	HR (L/min)	CO (L/min)	P_driving_ (cmH_2_O)	VT (mL)	RR (L/min)
START	PCV	67 ± 16	219 ± 10	0. 51 ± 0.18	5. 4 ± 0.8	24. 2 ± 1.7	22. 2 ± 1.6
	VV	68 ± 15	219 ± 21	0. 47 ± 0.12	5. 7 ± 0.7	24. 1 ± 1.6	22. 2 ± 1.6
CP	PCV	76 ± 14^a^	215 ± 20	0. 59 ± 0.18	8. 9 ± 1.1^a^	24. 1 ± 1.6	22. 4 ± 1.4
	VV	78 ± 14^a^	215 ± 31	0. 54 ± 0.14	8. 5 ± 1.5^a^	24. 1 ± 1.5	22. 1 ± 1.6
post-CP	PCV	75 ± 13	225 ± 11	0. 58 ± 0.16	5. 2 ± 0.8^b^	23. 8 ± 1.7	22. 4 ± 1.4
	VV	73 ± 15	225 ± 20	0. 53 ± 0.18	5. 3 ± 0.8^b^	24. 2 ± 1.7	22. 2 ± 1.6

Measurements were made 15 min after initiating each ventilation mode (START), 15 min after abdominal insufflation with CO_2_ (CP), and 15 min after deflation of the abdomen (post-CP).

Abbreviations: CO, cardiac output; HR, heart rate; MAP, mean arterial pressure; PCV, pressure-controlled ventilation mode; *P*_driving_, driving pressure; RR, respiratory rate; SD, standard deviation; VT, tidal volume; VV, variable ventilation.

a *P* < .05 vs START within a ventilation mode.

b *P* < .05 vs CP within a ventilation mode. Applied statistical test: 2-way RM ANOVA.

**Figure 4.**
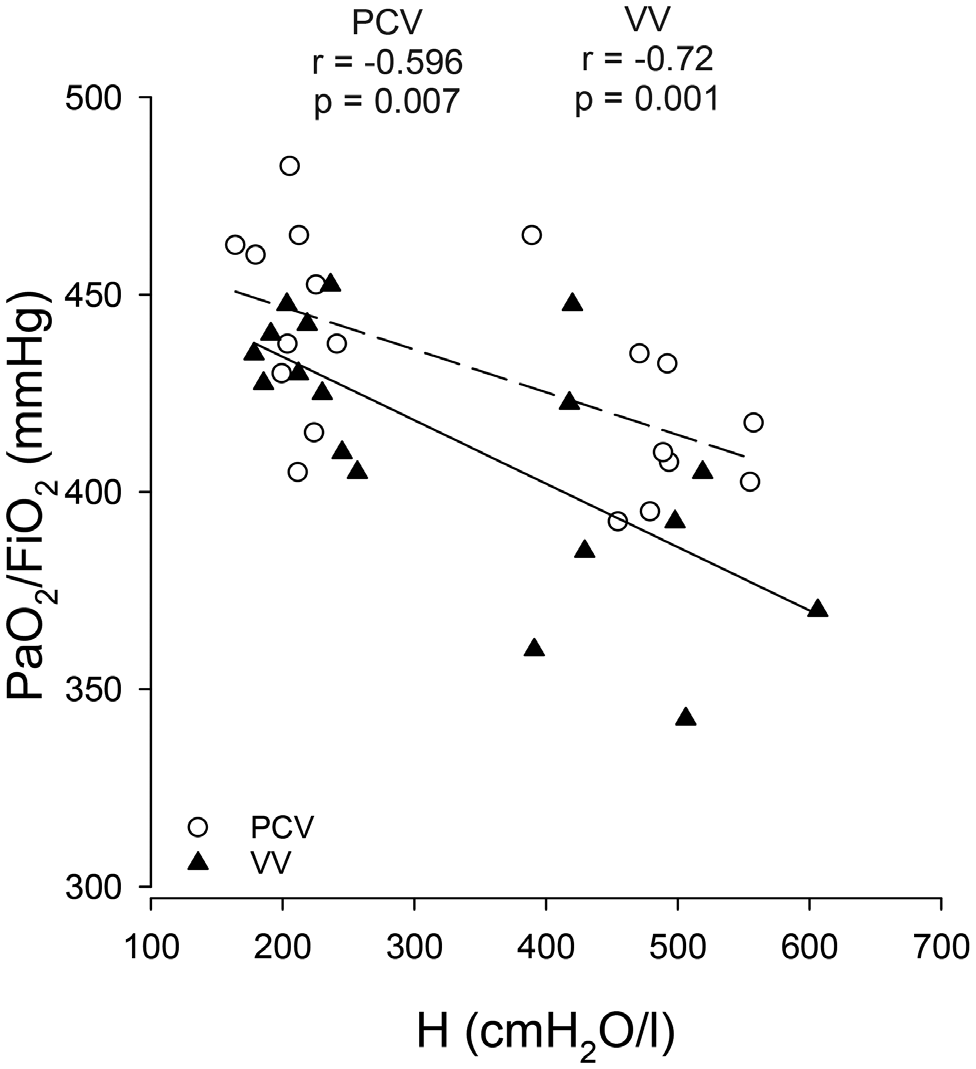
Correlation analyses between lung oxygenation index (Pao_2_/FiO_2_) and respiratory elastance (H) under different ventilation modes and interventions. PCV indicates pressure-controlled ventilation; VV, variable ventilation.

To explore the associations between the alterations in the lung oxygenation index (Pao_2_/FiO_2_) and respiratory elastance (H) under different ventilation modes and interventions, their relationship is depicted in Figure 4. There is a significant correlation between these respiratory tissue mechanical and gas exchange outcomes during ventilation with both VV and PCV modalities.

## DISCUSSION

In the present study, a well-established capnoperitoneum model^26,27^ was applied to explore the potential benefit of a VV pattern on the compromised gas exchange, and to compare this with conventionally used PCV. Abdominal insufflation with CO_2_ compromised gas exchange and respiratory mechanics under pressure-controlled and variable mechanical ventilation modalities. VV provided less efficient gas exchange under capnoperitoneum compared to conventional pressure-controlled mode. To mimic a clinical scenario after capnoperitoneum termination by deflating the abdomen, a hyperinflation maneuver was applied. Overall, the airway and respiratory tissue mechanical parameters returned to their baseline values. However, 15 minutes after releasing capnoperitoneum, the gas exchange outcomes showed some temporal differences, with normalized lung oxygenation but an incomplete recovery for the elimination of Paco_2_ due to the residual CO_2_ diffusion from the tissues. During this period, the variability in the VT and respiratory rate improved the elimination of CO_2_ compared to the conventional pressure-controlled modality.

Laparoscopic surgery involves CO_2_ insufflation into the abdominal cavity to increase visibility and access for the surgeons to the intraabdominal structures. This results in an increased intraabdominal pressure that induces a cephalic shift of the diaphragm predisposing the lungs to atelectasis formation in the basal regions. In agreement with previous results,^26^ this intervention led to a deterioration in respiratory tissue damping and tissue elastance (Figure 3.). Proportional increases in tissue damping and tissue elastance resulted in a constant η. Elevations in η is a well-established indicator of ventilation heterogeneities. The lack of change in this mechanical outcome therefore suggests a homogeneous derecruitment of basal lung regions. Furthermore, lung volume loss impaired gas exchange, as demonstrated by decreased Pao_2_/FiO_2_ and elevated Paco_2_ and Qs/Qt (Figure 2). Moreover, the supplemental abdominal CO_2_ load facilitated diffusion into the systemic circulation, thereby resulting in marked elevations in Paco_2_ (Figure 2).

An optimal ventilation strategy during capnoperitoneum involves adjusting ventilation parameters to provide lung protective ventilation and ensure adequate CO_2_ clearance. This approach would necessitate an increase in RR and/or increase in P_driving_ to maintain a constant VT. The application of a VV pattern could be an alternative lung protective strategy in the presence of capnoperitoneum, owing to its previously described benefits in restrictive lung disorders. In a lung fibrosis model, the ability of VV to prevent lung heterogeneity and to improve gas exchange was demonstrated.^14^ Interestingly, the results of the present study demonstrated no clear benefit of VV in terms of oxygenation and CO_2_ clearance during capnoperitoneum compared to conventional PCV; in fact, it even slightly worsened blood gas parameters. This seemingly controversial finding might be explained by homogenous lung volume loss in the basal regions rather than heterogenous lung derecruitment during abdominal insufflation.^28^ With continuous positive intraabdominal pressure, the recurring lower VTs during VV could lead to transitory alveolar collapse, not counteracted by occasional higher tidal inspirations. Accordingly, our findings suggest that during a persistent restrictive lung condition that developed during capnoperitoneum, the continuous external mechanical load on the alveolar compartment facilitates temporary alveolar collapse in the basal lung regions, which cannot be sustainably reopened by the recurring excessive lung expansions during VV. While the number of open alveoli during the ventilation cycle is directly related to the efficiency of gas exchange, these phenomena may not be reflected in differences in H, because the respiratory mechanical parameters were measured at end-expiration.

The relationship of conventional PCV and the advanced VV modalities in terms of their gas exchange benefits fundamentally changes on release of the capnoperitoneum. All respiratory mechanical parameters and the Pao_2_/FiO_2_ normalized after removing the mechanical load from the basal lung regions and performing alveolar recruitment, suggesting a complete recruitment of the previously collapsed lung areas. Nevertheless, detectable levels of hypercapnia, indicated by the significantly elevated Paco_2_ levels, remained. This may be attributed to residual CO_2_ diffusion from the tissues that were not eliminated after capnoperitoneum was released. Under these conditions, application of VV for 15 minutes showed a clear benefit in facilitating CO_2_ clearance and diminishing intrapulmonary shunting, thereby reducing Paco_2_ and Qs/Qt levels. Accordingly, the gas exchange benefit of VV can be manifested when the constant mechanical load is alleviated from the basal lung compartment, similar to its proven benefits in other lung disorders.^11,13–19^ This finding may also suggest the potential of VV to facilitate faster recovery from hypercapnia after capnoperitoneum. The improved CO_2_ clearance observed under VV is in accordance with the literature. VV has been shown to be beneficial to stabilize Paco_2_ and improve CO_2_ clearance in a preclinical bronchoconstriction model,^29^ in a chronic obstructive pulmonary disease model,^13^ a model of acute lung injury^30^ and in a model of acute respiratory distress syndrome^31^ compared to conventional ventilation modes.

A few limitations of the present study warrant consideration. First, in accordance with the 3R principles the research was conducted using only 1 study group, necessitating a crossover design. While this design helps control for some variables by allowing each subject to serve as its own control, it may also introduce potential biases and may limit the generalizability of the findings. Second, to minimize the effects of sex as a biological variable on the results of the present study, only male animals were used. However, a previous study using the same animal model of capnoperitoneum evidenced no differences in gas exchange parameters between sexes,^26^ suggesting the generalizability of our findings. Lastly, the duration of the different ventilation stages was restricted to 15 minutes each. Although these periods were long enough to reach a steady-state ventilation condition in healthy lungs, this relatively short timeframe may not be sufficient to fully reveal the differences between ventilation modalities during capnoperitoneum. These limitations suggest that further research, including a more diverse study group, longer observation periods, and comprehensive lung function assessments, are necessary to validate and expand the findings of this study.

In conclusion, this study investigated the effects of VV versus PCV during capnoperitoneum and the subsequent recovery phase. The findings indicate that while VV did not offer superior gas exchange compared to a conventional modality during capnoperitoneum, it proved beneficial in enhancing CO_2_ clearance after the release of the abdominal insufflation. Specifically, VV facilitated faster recovery from hypercapnia, most likely due to its ability to improve ventilation-perfusion matching once the constant mechanical load on the lungs was removed. These results highlight the potential utility of VV in clinical settings postcapnoperitoneum, although VV is unlikely to offer better gas exchange during the insufflation phase.

## ACKNOWLEDGMENTS

The authors thank Xavier Belin for his expertise in the handling and instrumentation of the study animals at our laboratory.

## DISCLOSURES

**Conflicts of Interest:** None. **Funding:** None. **This manuscript was handled by:** Christina M. Pabelick, MD.
